# Intracranial Venous Alteration in Patients With Aneurysmal Subarachnoid Hemorrhage: Protocol for the Prospective and Observational SAH Multicenter Study (SMS)

**DOI:** 10.3389/fsurg.2022.847429

**Published:** 2022-04-05

**Authors:** Giuseppe E. Umana, S. Ottavio Tomasi, Paolo Palmisciano, Gianluca Scalia, Valerio Da Ros, Rahman Al-Schameri, Stefano M. Priola, Lara Brunasso, Giuseppe Roberto Giammalva, Federica Paolini, Roberta Costanzo, Lapo Bonosi, Rosa Maria Gerardi, Rosario Maugeri, Lidia Strigari, Philip E. Stieg, Giuseppe Esposito, Michael T. Lawton, Christoph J. Griessenauer, Peter A. Winkler

**Affiliations:** ^1^Department of Neurosurgery, Trauma Center, Gamma Knife Center, Cannizzaro Hospital, Catania, Italy; ^2^Department of Neurological Surgery, Christian Doppler Klinik, Paracelsus Medical University, Salzburg, Austria; ^3^Laboratory for Microsurgical Neuroanatomy, Christian Doppler Klinik, Salzburg, Austria; ^4^Department of Neurosurgery, Highly Specialized Hospital of National Importance “Garibaldi”, Catania, Italy; ^5^Diagnostic Imaging Unit, Department of Biomedicine and Prevention, University of Rome “Tor Vergata”, Rome, Italy; ^6^Division of Neurosurgery Health Sciences North, Northern Ontario School of Medicine, Sudbury, ON, Canada; ^7^Post-graduate Residency Programme in Neurological Surgery, Department of Experimental Biomedicine and Clinical Neuroscience, School of Medicine, Neurosurgical Clinic, AOUP “Paolo Giaccone”, Palermo, Italy; ^8^Department of Medical Physics, IRCCS University Hospital of Bologna, Bologna, Italy; ^9^Department of Neurosurgery, Weill Cornell Medicine, New York, NY, United States; ^10^Department of Neurosurgery, University Hospital Zurich, University of Zurich, Zurich, Switzerland; ^11^Departments of Neurosurgery and Neurobiology, Barrow Aneurysm and AVM Research Center, Barrow Neurological Institute, Phoenix, AZ, United States

**Keywords:** brain aneurysm, brain circulation, endovascular coiling, subarachnoid hemorrhage, surgical clipping, vasospasm, venous alteration

## Abstract

**Background:**

Arterial vasospasm has been ascribed as the responsible etiology of delayed cerebral infarction in patients with aneurysmal subarachnoid hemorrhage (aSAH), but other neurovascular structures may be involved. We present the protocol for a multicenter, prospective, observational study focused on analyzing morphological changes in cerebral veins of patients with aSAH.

**Methods and Analysis:**

In a retrospective arm, we will collect head arterial and venous CT angiograms (CTA) of 50 patients with aSAH and 50 matching healthy controls at days 0–2 and 7–10, comparing morphological venous changes. A multicenter prospective observational study will follow. Patients aged ≥18 years of any gender with aSAH will be enrolled at 9 participating centers based on the predetermined eligibility criteria. A sample size of 52 aSAH patients is expected, and 52 healthy controls matched per age, gender, and comorbidities will be identified. For each patient, sequential CTA will be conducted upon admission (day 0–2), at 7–10 days, and at 14–21 days after aSAH, evaluating volumes and morphology of the cerebral deep veins and main cortical veins. One specialized image collecting center will analyze all anonymized CTA scans, performing volumetric calculation of targeted veins. Morphological venous changes over time will be evaluated using the Dice coefficient and the Jaccard index and scored using the Boeckh–Behrens system. Morphological venous changes will be correlated to clinical outcomes and compared between patients with aSAH and healthy-controls, and among groups based on surgical/endovascular treatments for aSAH.

**Ethics and Dissemination:**

This protocol has been approved by the ethics committee and institutional review board of Ethikkommission, SALK, Salzburg, Austria, and will be approved at all participating sites. The study will comply with the Declaration of Helsinki. Written informed consent will be obtained from all enrolled patients or their legal tutors. We will present our findings at academic conferences and peer-reviewed journals.

**Approved Protocol Version and Registration:**

Version 2, 09 June 2021.

## Introduction

Subarachnoid hemorrhage represents one of the deadliest type of strokes, and significantly burdens healthcare across the globe. As estimated, it accounts for 5–10% of all strokes, and, when non-traumatic, it mostly originates from ruptured intracranial aneurysms (1). The reported incidence of aneurysmal subarachnoid hemorrhage (aSAH) is highly variable worldwide, ranging from 3 to 23 cases per 100,000 persons/year, probably due to the geographic differences in genetic and/or social risk factors (2, 3). aSAH most commonly occurs in young individuals, with reported fatality rates of 25–50%- However, since a significant portion of patients die before receiving medical assistance, the actual death rate is expected to be higher (4, 5). In addition, aSAH strongly affects quality-of-life, as up to half of survivors suffer from long-term neurocognitive deficits and psychological symptoms, failing to fully return to work or independent life (6, 7). The modern management of aSAH remains focused in combining surgical/endovascular techniques with advanced neurocritical care and rehabilitation protocols, aimed at reducing mortality and neurological/systemic sequelae (8).

Delayed cerebral infarction (DCI) is a major cause of disability and mortality in patients with aSAH. DCI may occur in up to one third of patients, presenting with new focal neurological deficits and clinical deterioration 4–14 days after aneurysm rupture (9). The etiology of DCI has been historically ascribed to the transient narrowing on intracranial arteries (vasospasm) detected at angiography. Likely originating from the release of spasmogenic agents following subarachnoid blood degradation, arterial vasospasm frequently starts 3–4 days after aSAH and self-resolves by 14–21 days (10). However, the absolute relationship between vasospasm and DCI has been recently questioned due to the absence of DCI in half of patients with angiographically proven vasospasm and, in some cases, the discrepancy between narrowed vessels and ischemic territories (11, 12). This hypothesis has been further corroborated by several studies analyzing the prophylactic role of the nimodipine in preventing DCI. Despite reducing the risk of DCI and improving clinical outcomes, nimodipine showed no effects in altering the incidence and severity of arterial vasospasm (13). Hence, other than arterial vasospasm, a variety of neural and vascular changes induced by aSAH may concur in the onset of DCI.

In order to maintain the physiologic cerebral circulation, arterial blood inflow and venous outflow need to harmonize (14). By disrupting this circulation, a stroke event, such as aSAH, alters the vascular neural network and damages the neural tissue. Indeed, the aftermath of aSAH releases several oxidative stress factors and inflammatory cytokines, inducing arterial injury with brain edema. Consequently, the increased intracranial pressure compresses the thin wall of venules, slowing venous outflow and aggravating the oxidative stress and inflammation. At the same time, the large cerebral venous system appears to be also directly involved in aSAH. Dai et al. (15) demonstrated post-aSAH cerebral venous spasm in rabbits on MRI scans, noticing peaks at 5–7 days after aSAH with severely reduced venous blood flow leading to brain venous infarcts. Hence, in the presence of venous vasospasm and reduced venous drainage, arterial vasodilation may not be sufficient to improve venous flow and outcomes, likely explaining the limited results obtained using calcium-channel blockers for treating DCI compared to prevention (11, 16).

In humans, the relationship between aSAH and cerebral veins has not been thoroughly investigated. The potential role of large vein vasospasm needs to be examined, with the hope of improving treatment strategies for DCI, protecting and restoring the vascular neural network, and, thus, reducing morbidity and mortality in affected patients (14). Therefore, we present a multicenter, prospective, observational study, called Subarachnoid hemorrhage Multicenter Study (SMS), aimed at analyzing and quantifying potential morphological changes in cerebral and internal jugular veins following aSAH using sequential CT angiography (CTA) scans, including arterial and venous phases, obtained at patient admission and at later days after aSAH.

## Methods and Analysis

### Study Objective

We aim to detect and quantify potential morphological changes of cerebral and internal jugular veins in patients with aSAH using CTA scans performed at admission and at subsequent follow-ups. We also plan to examine possible correlations between the severity of venous vasospasm and clinical outcomes.

### Study Design

**Figure 1:**
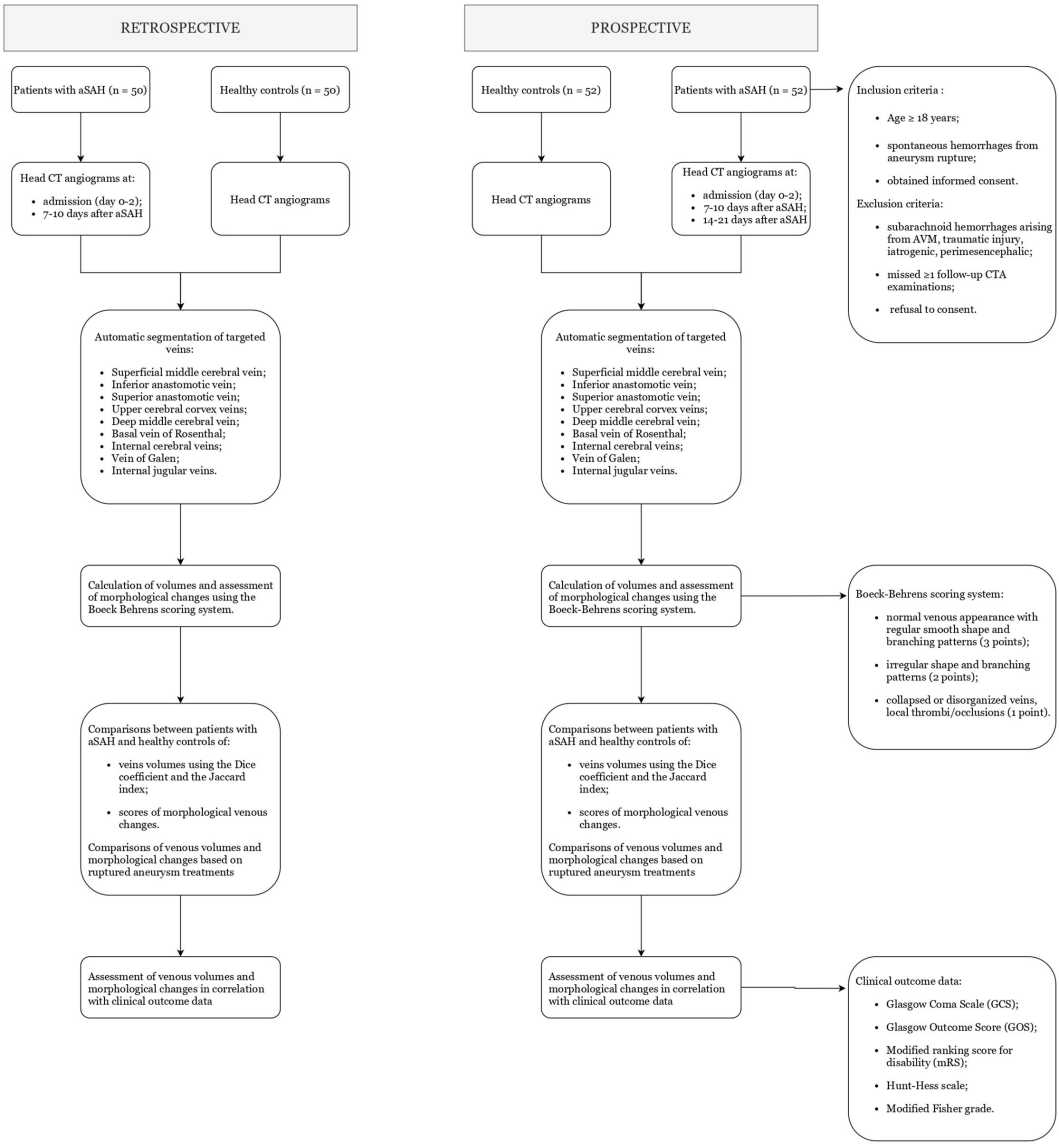
Flow chart of the study process.

In a retrospective arm of the study, we will collect head CTAs – both arterial and venous phases – of 50 patients affected by aSAH at day 0–2 and 7–10, and 50 matching healthy controls. CTA images will be analyzed and compared following the methodology described below. Based on these findings, we will move forward to a multicenter prospective observational study as follows. Nine centers from 3 countries will participate, ensuring adequate aSAH patient enrollment. Each enrolled patient will be followed-up for 6 months. The protocol has been approved by the local committee at the coordinating center (Department of Neurological Surgery Christian Doppler Klinik Paracelsus Medical University Salzburg, Austria) and is expected to be approved at each participating site. All participating centers will treat patients with aSAH according to local emergency and intensive care protocols and surgeon's expertise and will obtain standard CTA scans – comprising both arterial and venous phases – at each stage of care. Images will be anonymized and then sent to an image collection center (Sant'Orsola University Hospital, Bologna, Italy), which will segment, measure, and assess the morphological changes of cerebral and internal jugular veins. Figure 1 summarizes the study process algorithm.

The Department of Neurosurgery at Cannizzaro Hospital Trauma Center Gamma Knife Center in Catania, Italy and the Department of Neurological Surgery Christian Doppler Klinik, Paracelsus Medical University, Salzburg, Austria centers designed and launched the study, coordinated by the primary investigators (G.E.U., O.S.T., G.E., C. G., P.A.W). Additional 7 centers have been invited to participate in the study for improving the generalizability of the results, with each center having significant expertise and resources in the management of patients with aSAH. At each participating center, each participating center, a primary data collection recorder (neurosurgeon/neuroradiologist) and a data checker (neurosurgeon/neuroradiologist) were appointed. At this point we also welcome additional centers meeting the relevant standards to join. This protocol was drafted in accordance with the Standardized Protocol Items: Recommendations for Observational Studies statement (SPIROS) (Supplementary File 1).

### Patient and Public Involvement

Patients and the public were not involved in the study design. Patients will participate during the recruitment stage of the study on a voluntary basis. The study results will be disseminated to patients and the public through academic conferences and peer-reviewed scientific publications.

### Population and Recruitment

The analysis of the retrospective cohort is planned to start in 2022. The recruitment of prospective patients will start after the statistical analysis of the retrospective cohort, and will end ~1 year later, with an anticipated sample size of 52 patients. The last 6-month follow-up assessment of the last enrolled patient is expected to be concluded by the fall of 2023. Patients aged ≥ 18 years of any gender presenting to the emergency room of participating centers with a confirmed diagnosis of aSAH will be eligible for inclusion. Patients will be recruited only if the inclusion criteria are met per the referring neurosurgeon/neuroradiologist at each participating center. Enrolled patients will be grouped based on the different ruptured aneurysm treatments, and differences in volumetric scores will be analyzed separately for each of the following treatment groups: surgical clipping; endovascular coiling; other endovascular adjuncts (balloon-assisted coiling, stenting, flow-diverting stents); no treatment. An equal number of healthy patients, aged ≥18 years, not affected by aSAH or any other brain disease, and matched for age, gender, and comorbidities with the study participants, will be included in the control group. Healthy controls will be enrolled among hospitalized patients undergoing CTA examination for suspected vascular lesions but showing no pathological alteration at final CTA scans.

### Eligibility Criteria

For the aSAH cohort, the following inclusion criteria were set: (1) patients aged ≥ 18 years; (2) medical history of spontaneous hemorrhages from aneurysm rupture (aSAH); (3) obtained informed consent for study participation either from the patient or the next of kin.

The following exclusion criteria were set: (1) patients with subarachnoid hemorrhages arising from AVM, traumatic injury, previous brain surgery, perimesencephalic; (2) missed ≥1 follow-up CTA examinations; (3) refusal to consent.

### Outcome Measures

The primary outcomes of interest are the progressive morphological changes of cerebral and internal jugular veins detected at CTA examinations. The following veins will be studied: (1) the superficial middle cerebral vein (or superior Sylvian vein); (2) the vein of Labbé (or inferior anastomotic vein); (3) the vein of Trolard (or superior anastomotic vein); (4) other prominent veins of the upper cerebral convexity; (5) the deep middle cerebral vein; (6) the basal vein of Rosenthal; (7) the internal cerebral veins; (8) the vein of Galen; (9) the internal jugular veins. CTA scans will be acquired upon the following standardized protocol: (1) initial arterial phase with “Smart Prep” at the level of the aortic arch; (2) first venous scan obtained after 6.6 seconds (i.e., the minimum time delay to restart the volumetric acquisition); (3) second venous scan obtained 6.6 seconds after the firs venous scan. At each participating center, CTA scans will be performed as per standardized protocols at three different stages: at patient admission (0–2 days after aSAH); at 7–10 days after aSAH; and at 14–21 days after aSAH (17). Additional CTA scans will be obtained in healthy patients aged ≥ 18 years (i.e., not suffering from aSAH or any other brain diseases) and included in the control group. All morphological analyses will be conducted at the specialized image collection center (Sant'Orsola University Hospital, Bologna, Italy). For each CTA scan obtained at the different time intervals, the target veins will be segmented using an automatic tool. This algorithm is also capable of removing clips, coils, or other endovascular adjuncts from the field of view (FOV) of post-procedural CTA scans. For external validation, contours will be appraised by the referring physicians of each participating center. Volumes of segmented veins will be calculated after contrast administration, for ~1 cm of their length including the section with abnormal morphology detected by the referring neuroradiologist at each participating center. The venous morphology will also be evaluated using the scoring system proposed by Boeckh–Behrens et al. (18), defining: normal venous appearance with regular smooth shape and branching pattern (3 points); irregular shape and branching pattern of the veins (2 points); collapsed or disorganized veins, local thrombi/occlusions (1 point). The volumes and morphological scores of the segmented veins will be compared between patients with aSAH and healthy controls. The baseline CT dataset of the prospective arm will be compared with the control's mean value CT, and the prospective cohort's second and third CT datasets will be compared with the baseline CT dataset of the same prospective cohort.

The secondary outcomes of interest are correlations of venous morphological scores with clinical outcomes in patients with aSAH. Clinical outcomes will be calculated at patient admission and subsequent follow-ups using the following scoring systems: Glasgow Coma Scale (GCS), Glasgow Outcome Score (GOS), modified ranking score for disability (mRS), World Federation of Neurological Surgeons (WFNS) SAH grading, the Hunt–Hess scale, the modified Fisher grade, and the BNI SAH grading scale. At each participating center, such measurements will be completed by one senior neuroradiologist and one senior neurosurgeon, each with ≥ 10 year of experience. For each patient, the following data on management courses will also be specified: presence of arterial vasospasm detected at CTA; completion of external ventricular drainage or other procedures for cerebrospinal fluid diversion; intensive care supports provided for hemodynamic and respiratory stabilization.

### Data Collection and Management

All anonymized data on patients' demographics, clinical variables, venous volumes, and morphological venous changes will be collected and stored in a digital data bank accessible to each referring physician involved in this study. At each participating center data will be entered into an online database by primary data collection recorders who obtained entry qualifications and regularly reviewed for quality control. The specialized image collection center (Sant'Orsola University Hospital, Bologna, Italy) will have access to anonymized data on CTA scans shared by participating centers but not any of the related clinical information. The coordination center (Department of Neurological Surgery, Christian Doppler Klinik, Paracelsus Medical University, Salzburg, Austria) will have access to all data and will ensure the correct management and operation of the data bank. At the end of the study, collected data will be analyzed and published in an anonymized form.

### Sample Size Calculation

The primary objective of our study is to investigate potential volume and morphological venous changes in patients with aSAH and compare venous scores to an equivalent cohort of healthy patients matched per age and gender. Contours of cerebral veins and jugular veins on three patients and three controls were measured in a pilot statistical study to calculate the required prospective sample size. Assuming a normal distribution of all obtained venous volumes, a two-tailed alpha level of 0.05, and a power of 0.8, we calculated that a minimum sample size of 52 patients in the aSAH cohort is required to reach a mean effect size, and an equal number of healthy controls needs to be identified.

### Data Analysis

All statistical analysis will be performed using R-package or Matlab (The Mathworks Inc. Natick, MA). Continuous variables, such as volumes of segmented veins, will be presented as means or medians and ranges, while categorical variables, such as scoring systems for venous morphology and clinical outcomes, will be presented as frequencies or percentages. Differences between patients in the aSAH cohort and healthy control, and among treatment groups, will be compared using the repeated-measures Student *t*-test or Mann-Whitney U test for continuous variables, and χ^2^ test or Fisher's exact test for categorical variables.

The volumes of the target veins, studied at different time intervals, will be compared using the Dice coefficient and the Jaccard index (19, 20). These indexes will be calculated using MIM Vista (MIM Software, Cleveland, OH) at the collection center. The Dice coefficient and the Jaccard index are similar statistical measures of the spatial overlap between two volumes and are calculated by the following equations: Dice coefficient: (A,B) = 2 |AnB||A|+|B| and Jaccard index: (A,B)=|AnB||A?B| (where A and B denote the volumes to be compared). The Dice coefficient is defined as 2 × intersection volume/total sum of volumes, while the Jaccard index describes the volume of intersection between two volumes/volume of the union of these volumes. Both overlap coefficients normalize the degree of intersection from 0 (no overlap) to 1 (perfect overlap). The comparison between patients and controls will be performed based on the contours drawn on the baseline CT images of patients and those of controls. In the patients' group, we will assess the contours change over time compared to the baseline determination.

## Discussion

This protocol describes the SMS study, which will be the first multicenter project exploring morphologic and volumetric changes of the cerebral venous system in patients with aSAH, from hospital admission to 21 days follow-up. In particular, the SMS study aims at scoring morphological venous changes following aSAH and analyzing their role in relation with severity of clinical outcomes and functional status of affected patients. We will also compare these pathological findings with venous morphology obtained from healthy individuals without aSAH nor other brain diseases.

The management of patients with aSAH remains challenging, and the primary goal focuses on preventing DCI or easing its clinical burden (8, 10). In addition to the substantial expenses of surgical/endovascular treatments of aSAH, delayed aSAH complications considerably increase costs of patient care due to the high morbidity and mortality rates in affected individuals (6, 21, 22). On the basis of the decreased cerebral perfusion and the narrowed arteries detected angiographically, several arterial vasodilator agents are currently adopted worldwide as feasible treatments; however, their clinical impact and outcome results are ambiguous (23). Still, the poor cerebral blood flow appears to be the primary factor in DCI, thus different structures of the neural vascular network have been hypothesized to be involved other than the cerebral arteries (14). In particular, the cerebral venous system may play a pivotal role in the aftermath of aSAH, since the decreased venous drainage may favor tissue ischemia by contrasting the removal of newly released inflammatory and oxidative substances (24, 25).

The glymphatic system, present within the para-vascular spaces in tight relation with astrocytes' aquaporin-4 channel, serves as a para-vascular cerebrospinal fluid stream having a bidirectional flow with input from para-arterial spaces and output to para-venular spaces through the aquaporin-4 channels (26). In the pathophysiology of post-SAH DCI, pericytes have been demonstrated to regulate the diameter of small vessel, such as capillaries, venules, and arterioles, via exchanges of oxygenated hemoglobin (OxyHb) between the para-vascular and glymphatic systems. Indeed, OxyHb suppresses nitric oxide and cyclic guanosine monophosphate molecules, leading to pericytes contraction and reduction of small vessels' calibers (27, 28). OxyHb also promotes the production of reactive oxygen species (ROS) and endothelin 1 (ET-1) in astrocytes, causing further increase in pericytes contraction and astrocyte swelling with greater reduction of vessels' lumen, contributing to the worsening of the normal blood stream (28–30). As a consequence of SAH, the reduction of the brain perfusion causes diffuse ischemia and greater intracranial pressure, further favoring the constriction of the pericytes with worsening of the blood circulation (31–33).

The role of pericytes in the regulation of the vessel's lumen is fundamental due to their anatomic and functional relation with the brain vessels. While pericytes shield postcapillary venules, larger venous vessels, like the cerebral veins, present stellate peri-endothelial cells on their surface, which play a similar role to the smooth muscles of the arterial vessels (34). Moreover, the cerebral venous system is regulated by adrenergic nervous system (35). Due to their physical properties, major and minor veins are likely to collapse in response to minor external stimuli including brain swelling, increase in intracranial pressure, and direct compression by clots. The compression of the veins causes a further increase in intracranial pressure due to the reduced blood outflow, starting a self-sustaining mechanism typical of the post-SAH pathophysiology (35). In literature, animal models have been reported, demonstrating the importance of the venous system alterations as a consequence of SAH functional and structural modifications. The injection of blood or the positioning of clots at the level of the subarachnoid space has showed to stimulate the infiltration of erythrocytes in the Virchow-Robin spaces with congestion of capillaries and other small vessels, which may even collapse and cause brain swelling (36). MRI studies performed in rats after clot delivery at the level of the cisterna magna have also showed marked rise of the venous volume at 3 hours and 1 day after SAH (37). Another study on rabbits described vasospasm or collapse in deep cerebral veins due to brain swelling compression, with an increasing rate starting from day 1 and progressively worsening until day 5–7 (31). Finally, endothelial venous damage is the result of inflammatory process and oxidative stress, and this can favor clot formation in the venous system as a consequence of SAH, with high risk of thrombosis due to the slower blood flow at this level (35, 38).

However, clinical and radiologic data on volumetric and morphological changes of cerebral veins following aSAH are lacking in the current literature. With the SMS study, we aim to gather comprehensive data on the role of cerebral venous system in patients with aSAH and validate a standardized protocol for radiologic venous assessment in routine clinical settings. Indeed, despite digital subtraction angiography remaining the gold standard for aSAH, we prefer to use CTA due to its favorable accessibility, reduced invasiveness and costs compared to other radiologic modalities (39). Then, by relating morphologic venous changes, if any detected, to the clinical status of patients with aSAH, we may be able to identify favorable treatments, medical or endovascular, for improving cerebral venous outflow and functional outcomes (40).

The main limitations of this study, similarly to other multicenter prospective observational studies, will mostly comprise of differences in treatment approaches for aSAH among participating centers and the loss of follow-ups in some patients. While the heterogeneity in aSAH treatments will likely be of limited importance for the outcomes of this study which mostly focuses on radiologic assessments, we plan to overcome the limitations related to lost follow-ups by planning to contact enrolled patients via email or phone. Lastly, we will exclude any patients with incomplete follow-up results at the end of the study.

In conclusion, this prospective study is expected to improve the current understanding of the role of the cerebral venous system in patients with aSAH. Hopefully this will help to optimize treatments and clinical outcomes of aSAH patients.

## Strengths and Limitations

Controversies currently exist about the absolute role of arterial vasospasm in DCI following aSAH, and the clinical interest is slowly steering toward a more comprehensive understanding of the neural vascular network.

This will be the first multicenter, prospective, observational study to evaluate and quantify the morphological venous changes in patients with aSAH and examine potential correlations with the severity of clinical outcomes.

The cerebral venous system will be investigated using standardized and worldwide accessible CTA examinations, with the hope to better define its role in the pathogenesis of DCI and guide new treatments aimed at improving the management of such a dire complication.

Some limitations of this study may be represented by variations in aSAH treatments based on individual expertise at the different participating centers, but the centralized volumetric analyses of CTA scans will likely prevent subjective biases in radiological assessments.

## Ethics and Dissemination

The SMS study protocol has been approved by the Medical Ethics Committee of Ethikkommission, SALK, Salzburg, Austria. At each participating center, ethics approval will be obtained by communication of the obtained ethical approval from the coordinator center (Department of Neurological Surgery, Christian Doppler Klinik, Paracelsus Medical University, Salzburg, Austria), to the local ethics committees before starting the patient recruitment. All patients will be provided with full oral and written information about the protocol and their involvement in the study. Before enrollment, signed consent forms will be obtained for each patient or the next of kin. Healthy individuals (i.e., not suffering from aSAH nor other brain diseases) comprising the control group will be selected at each participating center from registries of patients with no brain-related conditions and will be approached by the referring physicians to receive full information about their involvement in the study and to provide their signed written consent. All participating centers will share the results of this study, which will be presented at academic conferences and peer-reviewed scientific journals.

## Ethics Statement

This protocol has been approved by the Ethics Committee and institutional review board of Ethikkommission, SALK, Salzburg, Austria, and will be approved at all participating sites. The study will comply with the Declaration of Helsinki. Written informed consent will be obtained from all enrolled patients or their legal tutors.

## Author Contributions

GU and ST are the primary investigators, proposed and initiated SMS, and defined the research strategy. PP, GU, PW, GE, and CG contributed substantially to conception, design of the study, and drafting of the manuscript. ML, LS, VD, GS, RA-S, PS, and GE helped to draft the manuscript and revised the manuscript for important intellectual content. RM and LB visualization and final draft validation. GG and RG data collection and first draft editing. FP did statistical analysis. RC did data curation and visualization. LB language revision and first draft editing. All authors have read and approved the final manuscript.

## Conflict of Interest

The authors declare that the research was conducted in the absence of any commercial or financial relationships that could be construed as a potential conflict of interest.

## Publisher's Note

All claims expressed in this article are solely those of the authors and do not necessarily represent those of their affiliated organizations, or those of the publisher, the editors and the reviewers. Any product that may be evaluated in this article, or claim that may be made by its manufacturer, is not guaranteed or endorsed by the publisher.
