# Supplementary material for: Intracranial Venous Alteration in Patients With Aneurysmal Subarachnoid Hemorrhage: Protocol for the Prospective and Observational SAH Multicenter Study (SMS)
Source: Front Surg. 2022 Apr 5;9:847429. doi: 10.3389/fsurg.2022.847429 (PMC9018107; doi:10.3389/fsurg.2022.847429)
Supplement: Supplementary file 1 [file Table_1.DOCX]

Supplementary Table 1. Checklist of preliminary items based on Standard Protocol Items for Observational Studies (SPIROS).

| **Section and Topic** | **Description and Subcategories** | **Addressed on page number** |
| --- | --- | --- |
| 1. **General Information** | | |
| Title | Descriptive title identifying study design | Page 1 |
| Protocol version | Version or amendment number and date and summary of changes | Page 3 |
| Protocol summary | Brief summary of protocol research | Page 2-3 |
| Sponsor and partner  institute name | Name of sponsor and participating institutes (if applicable) | Pages 1 and 7 |
| Investigators’ names | Name of principal and co investigators. | Page 1 |
| Affiliation of  investigators | Affiliated institutions of investigators | Page 1 |
| Principal researcher  contact detail | Name, email address, affiliation of Principal researcher for correspondence. | Page 1 |
| Table of content | Table of content | Figure 1 |
| Page number | Page number on each page of protocol | Pages 5-13 |
| List of Abbreviations | A detailed List of all abbreviations used in protocol with full form. | NA |
| **ii) Introduction** | | |
| Background of study | Scientific background of study | Pages 5-6 |
| Review of prior  research | Summary of all previous relevant research | Page 6 |
| Rationale of study | Justification for conducting the study | Page 6 |
| Aim | Broader aims and specific objectives of the study | Pages 6-7 |
| Objective of study | Primary and secondary objectives of study | Page 7 |
| Prespecified  hypothesis | Prespecified null or alternative hypothesis | NA |

| **iii) Methods** | | |
| --- | --- | --- |
| Study design | Description of type/design of study | Page 7 |
| Study setting | Description of setting, locations, relevant dates, including periods of recruitment/survey, exposure, follow-up, and data collection.  Schedule of study procedure – Figure or table | Pages 7-11  Figure 1 |
| Sample size | Estimated number, calculation and assumptions  Power calculation | Page 8  Page 10 |
| Sampling procedure | Description of sampling strategy to ensure representativeness and control  of potential bias | Page 8 |
| Participants | Cohort study—eligibility criteria, and the sources and methods of  selection of participants. Describe methods of follow-up.  For matched studies, give matching criteria and number of exposed and unexposed  Case-control study—Give the eligibility criteria, and the sources and methods of case ascertainment and control selection. Give the rationale for the choice of cases and controls  For matched studies, give matching criteria and the number of controls per case  Cross-sectional study—Give the eligibility criteria, and the sources and methods of selection of participants | Pages 7-11 |
| Variables | All outcomes  Exposures- definition of exposure of interest,  Predictors  Potential confounders  Effect modifiers | Page 9 |
| Data Sources/ Measurement | For each variable of interest, give sources of data and details of methods of assessment (measurement).  Describe comparability of assessment methods if there is more than one group  Data collection points table  Blinding procedure | Pages 7-11  NA |
| Bias | Describe any efforts to address potential sources of bias  More specifically-  Information bias  Selection Bias  Control for confounding | Page 13 |

| Statistical analysis  plan | Method of primary / secondary outcomes and additional analysis  Handling of missing data  Post-hoc analysis | Page 11 |
| --- | --- | --- |
| Handling of withdrawals and lost to  follow up | Describe the procedures to be followed when a participant ceases participation in the study prematurely or is lost to follow up | Page 7 |
| Replacements | Provide information on whether or not participants who discontinue the  study will be replaced via additional recruitment to maintain the required sample size. | NA |
| Outcome | Define and describe all primary and secondary outcome or lost to follow  up | Page 9 |
| Database management | Detail plan of database management including:  Data collection (electronic or paper based),  Source data  Data entry  Data editing  Coding  Data storage  Record retention  Data confidentiality | Page 10 |
| Validation of  instrument | Reliability / validity of instrument or plan to establish validation | Page 11 |
| Follow up | Plan of follow up and addressing lost to follow up | Page 9 |
| Quality control | Method of quality control  Monitoring (internal and external)  Training of surveyors | Page 10 |
| Quality assurance | Plan of quality assurance | Page 10 |
| Expected outcome/results | A brief description of expected outcome or results | NA |

| **iv) Ethical consideration** | | |
| --- | --- | --- |
| Ethical approval | Weather it has been obtained and name of ethical committees. If approval  not sought, Reason | Pages 2-3 and 14 |
| Agreement and  consent | Method of taking consent. Reason if consent not sought | Pages 2-3 |
| Risk / Harm to  participants | Any potential risk or harm to study participants | NA |
| Adverse event and Severe adverse event  reporting | Outline how Adverse Event and Severe adverse event information will be collected. | NA |
| **v) Reporting and dissemination** | | |
| Protocol  amendments | Methods of communicating to investigators/IRBs and documenting | NA |
| Dissemination | How results will be disseminated to participants, practitioners, public | Pages 2-3 and 14 |
| Publication Plan | Who has right to publish; restrictions; authorship guidelines  Open Access | Pages 2-3 and 14 |
| Reporting of early  stopping | Dissemination of results if trial is stopped early (for any reason) | NA |
| **vi) Others** | | |
| Limitations | Limitations of proposed study, including risk of bias | Pages 4 and 13 |
| Strength of study | Highlight strengths of proposed study | Pages 4 and 13 |
| References | List of references cited in protocol | Pages 16-18 |
| Data collection  forms | Summary table of all forms used for data collection at each point of study | NA |
| Informed consent  forms | Sample of informed consent form, translated into local language | NA |
| Funding | Source of funding and the role of the funders for the present study | Page 15 |
| Acknowledgement  for protocol development | Acknowledgement of persons involved in protocol preparation | Page 15 |
| Data sharing policy | To describe how data will be made available in public domain. | Page 15 |
| Contributions of  authors to protocol | Listed authors should have participated sufficiently in preparation of  protocol with details of their contribution. | Page 15 |
| Trial registry | For observational studies also registered as trial | NA |
| Annexures | Data collection form /instruments Informed consent form  Standard operating procedures (SOPs) Detailed Statistical analysis plan (SAP) | NA |
